# Massive ovarian oedema: a misleading clinical entity

**DOI:** 10.1186/s13000-016-0469-3

**Published:** 2016-02-03

**Authors:** Nikolaos Machairiotis, Aikaterini Stylianaki, Paraskevi Kouroutou, Polixeni Sarli, Nikolaos Konstantinos Alexiou, Elias Efthymiou, Athanasios Maras, Nikolaos Georgios Alexiou, Spyridon Evaggelos Nikolaou, Nikolaos Courcoutsakis, Eleni Papakonstantinou, Paul Zarogoulidis, Nikolaos Barbetakis, Dimitrios Paliouras, Apostolos Gogakos, Christodoulos Machairiotis

**Affiliations:** Obstetric - Gynecology Department, “Thriassio” General Hospital of Athens, George Genimata, 19600 Athens, Greece; Trauma Surgery Department, “ Centre Hospitalier de Luxembourg”, General Hospital of Luxemburg, 4 Rue de Marble, Luxembourg, Belgium; 1st Internal Medicine Department, “Thriassio” General Hospital of Athens, George Genimata, 19600 Athens, Greece; Radiology Department, University General Hospital of Alexandroupolis, Democritus University of Thrace, Alexandroupolis, Dragana Greece; Pathology Department, “Thriassio” General Hospital of Athens, George Genimata, 19600 Athens, Greece; Pulmonary Department-Oncology Unit, “G. Papanikolaou” General Hospital, Aristotle University of Thessaloniki, Thessaloniki, Greece; Thoracic Surgery Department, Theagenio Cancer Hospital, Thessaloniki, Greece

**Keywords:** Massive ovarian oedema, Pedicle torsion, Luteinization of the stroma

## Abstract

**Background:**

Massive ovarian oedema is a rare non-neoplastic clinicopathologic entity has a higher incidence in women during their second and third life decade. The oedema can be presented in one or both ovaries as a result of partial intermittent torsion of the ovarian pedicle that interferes to the venal and lymphatic drainage of the ovary.

**Case presentation:**

We present a clinical case of a 16 year old with massive ovarian oedema and we performed a review of the literature. The pathophysiology of this entity is very complex. We tried to perform a complete review of the literature and focus on the complexity of this entity as far as its pathophysiological backround is concerned and as far as its clinical presentation is concerned.

**Conclusions:**

In conclusion, massive ovarian oedema is a rare, multi disease mimicking clinical entity, with an acute or progressive clinical presentation. It has also to be a part of our differential diagnosis in cases of acute abdominal pain and we have to try to treat her conservatively, in order to preserve fertility.

## Background

Massive ovarian oedema is a rare clinical entity, which is benign and has a higher incidence in women during their second and third life decade. This lesion was first described in 1967 [1] as a “tumour like” enlargement of the ovary due to oedema fluid. The oedema can be presented in one or both ovaries as a result of partial intermittent torsion of the ovarian pedicle that interferes to the venal and lymphatic drainage of the ovary [2]. The fact that the ovarian torsion is incomplete explains the fact that an ovarian cell necrosis is not caused; the effect of this lymphatic drainage obstruction is the enlargement of the ovary, which can be presented to the patient as a solid, adnexal mass. The clinical presentation of ovarian oedema can be either acute or progressive depending on the rapidity of the torsion. In other words, if the torsion is acute; the symptomatology is acute abdominal pain and could be mimicking an acute abdomen. If it presents in the right ovary and it’s expressed with pain in the right lower abdominal quadrant, has to be differential diagnosed from appendicitis. If the torsion is gradual then a stromal luteinization is provoked and the patient is often virilized. Menstrual irregularities, precocious puberty or even Meigs syndrome can be the clinical manifestations of this entity [3] (Table 1).

**Table 1:** Clinical findings upon admission and discharge

Clinical findings upon admission	Clinical findings upon discharge
Persistent abdominal pain in her right lower abdominal quadrant	Abdomen without any symptomatology
Palpable abdominal mass extending from the fossa iliaca to the liver	Abdomen soft, without peritonaism in palpation
CA 125 marker of 57,9	

*CT* cystic lesion 23 × 15 cm located in the right oblique abdominal area

## Case presentation

**Fig. 1:**
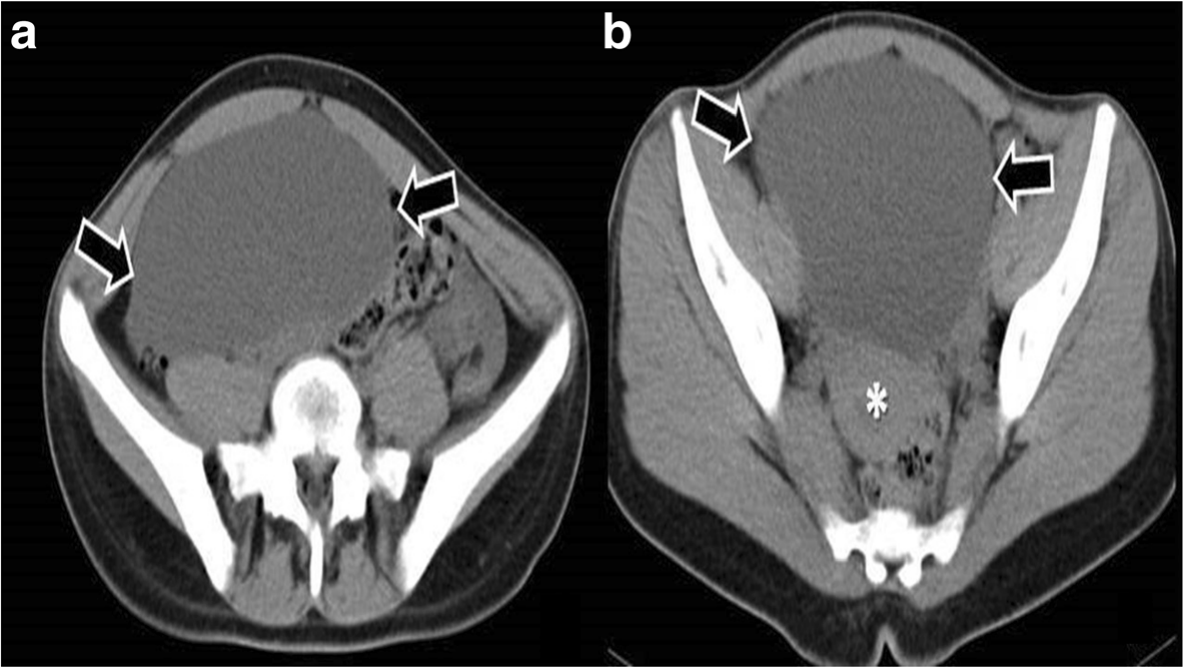
**a** and **b**: axial, unenhanced CT images of the pelvis demonstrate a voluminous mass [arrows] which displace the adjacent viscera [small intestine and uterus (star)]

**Fig. 2:**
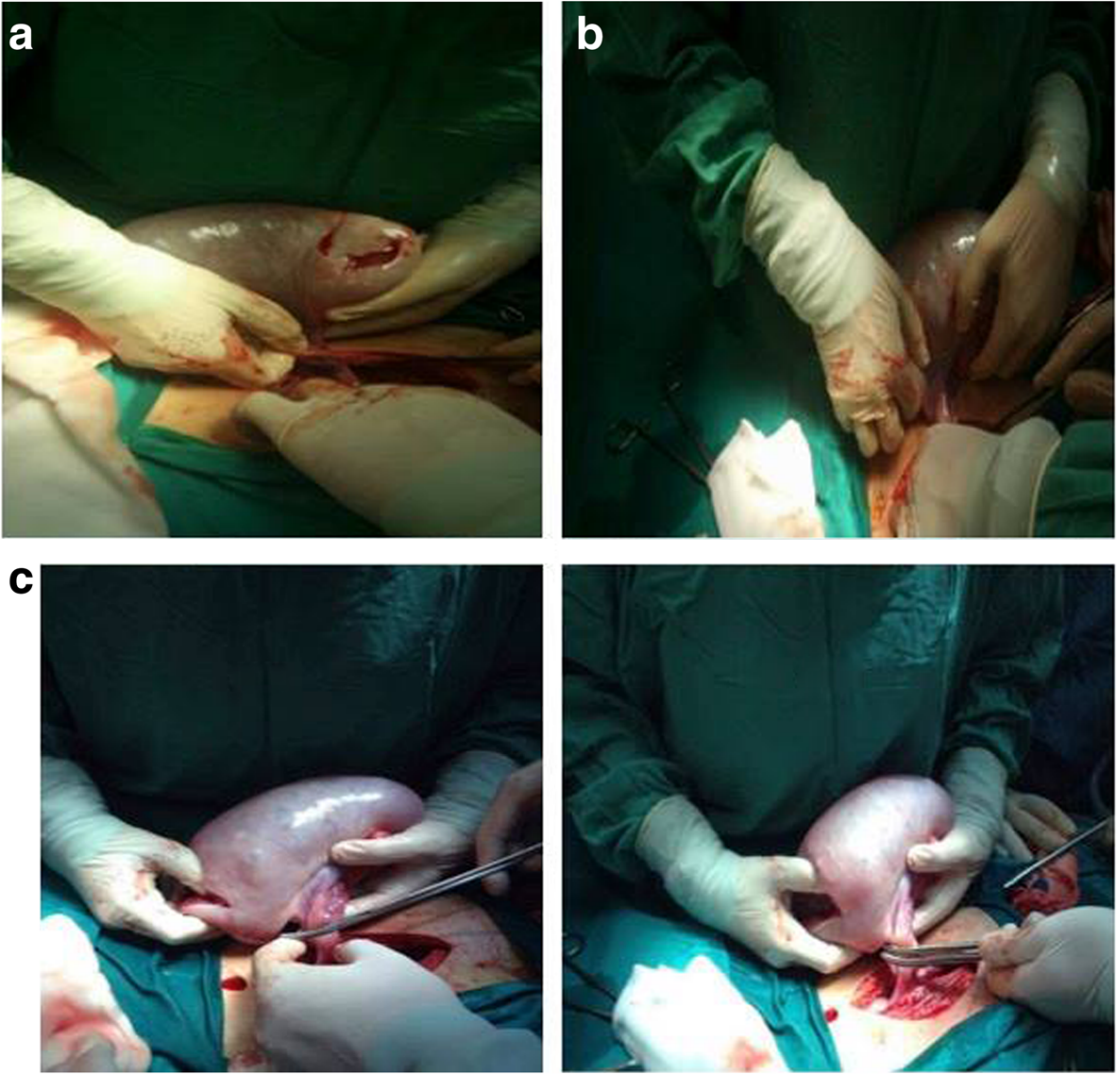
**a** incision of the ovary and triple torsion of the ovarian pedicle **b** anti-rotation of the pedicle – exclusion of possible ischemia or necrosis **c** (i)-(ii) excision of the mass

**Fig. 3:**
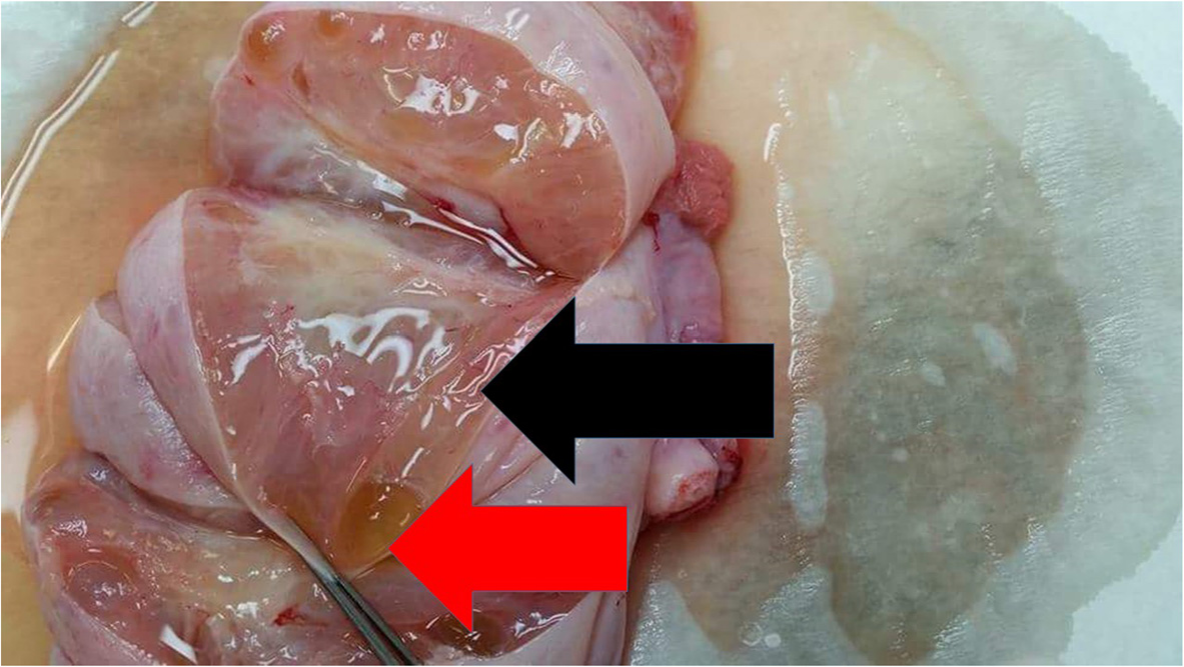
Red arrow; cystic lesions of the ovary, black arrow; stromal oedema

**Fig. 4:**
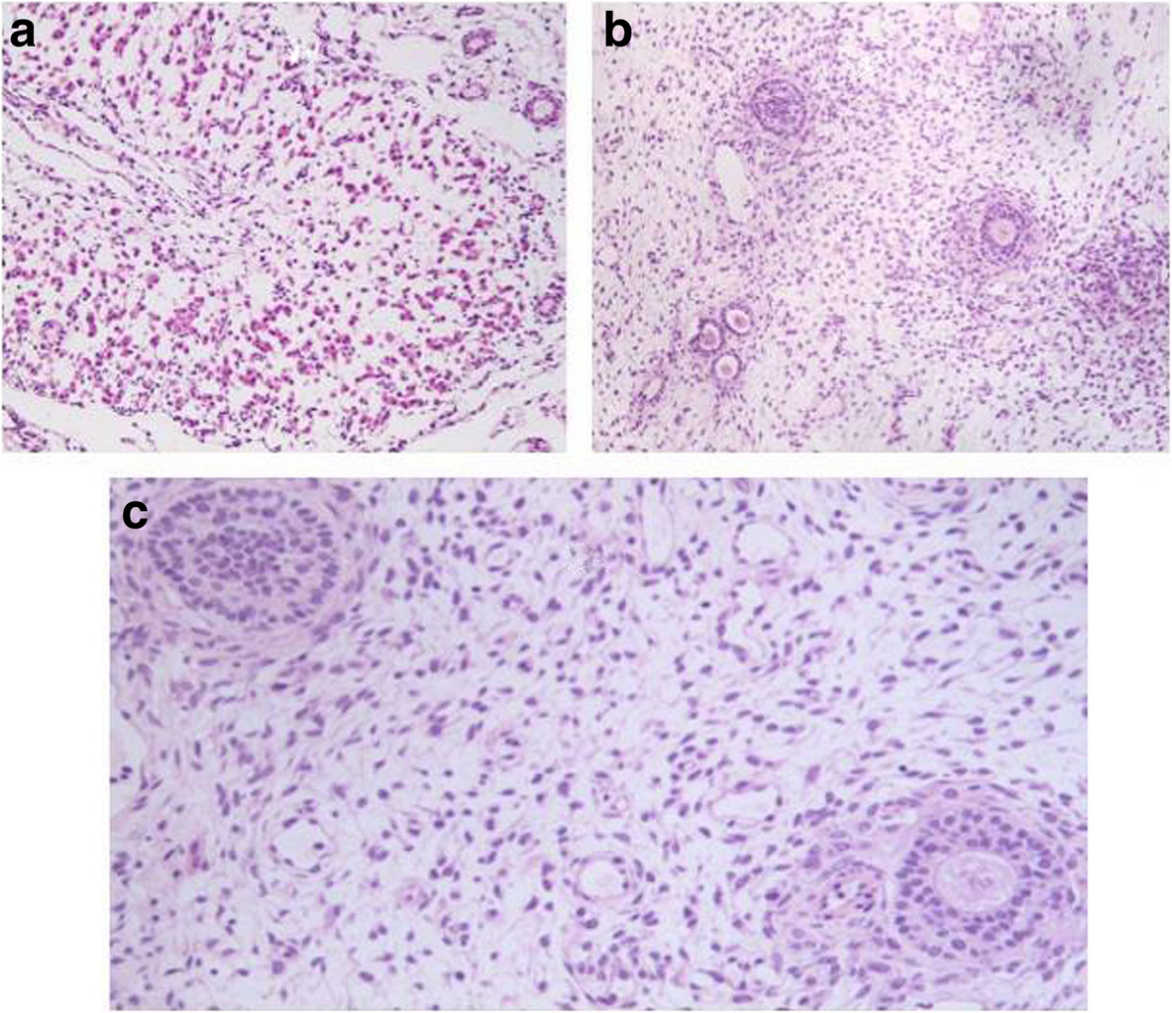
**a** cluster of lutein cells in the ovarian stroma **b** oedematous, fibroblastic stroma surrounding follicles and cluster of lutein cells on the left **c** οedematous, fibroblastic stroma surrounding follicles

A 16-year-old patient was admitted in our emergency department with persistent abdominal pain in her right lower abdominal quadrant. The patient claimed that the pain was colic like, persistent and gradually advancing the last 2 months. Her medical history was free, her menarche was in the age of 13 years and her menstrual cycle had a periodicity of 28 days. The clinical examination revealed a palpable abdominal mass extending from the fossa iliaca to the liver. The patient was hemodynamic stable. After two doses of intravenous paracetamol the pain was alleviated. We performed a radiologic and laboratory control; our imaging control included an Ultrasound of the lower abdomen and a Ct Scan of the upper and lower abdomen. An intravaginal ultrasound control could not be performed because the patient was still virgin. The CT scan revealed a cystic lesion 23x15cm located in the right oblique abdominal area, from the liver to the minor pelvis, which is causing pressure to the right kidney and the bladder and a small fluid collection in the Douglas region. (Fig. 1) This finding combined with the laboratory values, which showed an elevated CA 125 marker of 57,9 and a normal function of the hepatobilliary and the urinary system, led us to make a decision to perform a laparotomy. We performed a subumbilical incision of the abdomen. The ovary was identified, it was very swollen and there was fluid in the rightovarian pedicle. This specimen was taken for cytologic examination, which was negative for malignancy. An incision of the ovarian mass followed in order to make it possible to remove the ovary from the abdomen. The structure was bulky without any fluid and the excision was very difficult. Intraoperatively, after performing a light traction of the ovary a triple partial torsion of the ovarian pedicle was seen, nevertheless, there were no signs of ischemia or necrosis of the ovary. After careful preparation of the ovary and the pedicle we performed the excision and the specimen (excised ovary) was sent as a frozen section material for histopathologic examination (Fig. 2). The result was negative for malignancy and was compatible with normal ovarian tissue. The left ovary had a normal anatomy without any evidence of pathology or dysfunction. Macroscopically, the excided ovary was a kidney-like, very oedematous structure 23x14x5 cm. There was no sign of necrosis or hemorrhage, but there were many cystic lesions on the upper surface of the ovary, with a maximum diameter of 1.5 cm and a serous content (Fig. 3). Afterwards we performed an appendectomy. The patient recovered very fast postoperatively, without any pain and symptoms. The pathologic examination revealed a stroma oedema with presence of many fibroblast and collagen fibre, cystic ovarian folicules with lutenization in the periphery of the ovary without any stromal changes (Fig. 4). In the appendix many parasitic worms were present of the type of Enterobius Vermicularis.

## Discussion

Massive ovarian oedema is a tumor-mimicking condition occurring in young women [1]. It is often considered to be the result of complete torsion of the ovary to the extent that it interferes with venous and lymphatic drainage but is insufficient to cause necrosis [2, 4]. In addition to this mechanism, there is the pathophysiological mechanism of the partial ovarian torsion. This is based to the histopathological evidence of a hematoma [4]. There are many authors suggesting that partial torsion is a likely explanation for this perplexing disorder [4] and it considered to be a variant of polycystic ovary syndrome [5]. As a result of the enlargement of the ovary, the intrabdominal pressure rises, causing a pressure phenomenon in the nearby area and the patient usually presents with adnexal mass. The character of the symptomatology, acute pain or profound diffuse pain depends on the character of the torsion. In case of an acute torsion, the abdominal pain with the clinical presentation of an acute abdomen is the main symptom. In most of the cases described in the literature there are menstrual irregularities, infertility and abdominal distension [6]. Massive ovarian oedema is considered to be the result of ovarian lymphatic dysfunction. The question that rises is; how could the lymphatic dysfunction cause this kind of symptomatology? The answer is hidden in the unique capacity of the ovary to remodel its tissue structure and vascular network continuously and under a strictly controlled process.

The lymphatic vessels have a special morphology without a structural basement membrane and an overlapping layer of vascular endothelial cells. The functionality of these cells is controlled from the Vascular Endothelial Growth Factor (VEGF) and its receptor (VEGFR2) [7]. There are scientific studies showing that the inhibition of VEGFR2 prohibits the normal luteinization process [8, 9]. The ovarian lymph contains among others; hormones including progesterone, estradiol and inhibin that are transferred back to the ovarian arteries via retrograde transfer and then they promote the feedback in the hormonal regulation of the ovary [10, 11]. Given the mechanism mentioned above, the lymphatic dysfunction prohibits the normal luteinization of the ovary and causes hormonal problems. This is the mechanism that is thought to be responsible for the formation of primary ovarian oedema [12]. On the other hand massive ovarian oedema has been correlated in the literature with retroperitoneal lymphoma, metastatic carcinoma, polycystic ovary syndrome, metastatic-cervical carcinoma [12]. In those cases, massive ovarian oedema is characterized “secondary massive ovarian oedema” as a result of lymphatic vascular blockage. In patients under hormonal therapy and especially in therapy with clomiphene citrate the increase of LH and FSH cause changes in the lymphatic vasculature [13]. The menstrual irregularities can be a result of low serum levels of gonadotropins, because of an autonomous ovarian hormone production [14, 15]. This hormone production is a result of stromal luteinization according to Chervenak et al. [14]. On the other hand, Kalstone et al. suggested that the luteinization might be caused because of the mechanical stimulus of increasing quantity by oedema fluid which is stretching the stroma [1, 15]. Another theory for the formation of the oedema and the abnormal hormone production is the impact of insulin-like growth factor, epidermal growth factor or cytokines in the ovarian stroma cells [4]. There are very few literature references on massive ovarian oedema as a permeation of the ovarian lymphatics by metastatic carcinoma [16, 17]. The mechanisms mentioned above explain why masculanization and precocious puberty are common features among women suffering from massive ovarian oedema. The most interesting part of this clinical entity is the histopathological findings, which set the diagnosis. The ovarian stromal cells which are separated by copious oedema fluid with presence of atretic follicles without any involvement of the tunica albuginea and the superficial cortical zone are characteristically uninvolved [2]. A thin rim of compressed cortical stroma is recognized at the periphery of the mass. Necrosis and hemorrhage are unusual. Additionally, the presence of focal stream luteinization has also been described. The oedema of the stroma is thought to provoke the activation of fibroblasts and myofibroblasts in the stroma as a reaction to the oedema [18]. The therapeutic approach varies. The great majority of cases are unilateral and the most common treatment is unilateral salpingo-oopherectomy [19]. Frozen section is an option for preventing unnecessary catastrophic reproductive outcomes, on the other hand we have always to keep in mind the risk of recurrence [20]. Another option is wedge resection, which involves the removal of a minimum 30 % of the ovarian volume. This is performed in order to exclude secondary massive ovarian oedema. The possibility of postoperative adhesions is an argument to the complete removal of the ovary, because it provokes fertility issues [6, 21]. Laparoscopy can also be a therapeutic option for massive ovarian oedema as it combines diagnostics and therapy [22, 23]. Massive ovarian oedema is a result of the symptomatology and the intraoperative findings, as these lesions are often mistaken for primary ovarian neoplasms at laparotomy. Taking under consideration the age of the patients presenting with this entity, the preservation of fertility should be our first thought and conservative treatment must be the rule [19].

## Conclusion

Massive ovarian oedema is a rare, multi disease mimicking clinical entity, with an acute or progressive clinical presentation. It has also to be a part of our differential diagnosis in cases of acute abdominal pain and we have to try to treat it conservatively, in order to preserve fertility.

## Consent

Written informed consent was obtained from the patient for publication of this case report and any accompanying images. A copy of the written consent is available for review by the Editor-in-Chief of this journal.

## Abbreviations

LH: luteinizing hormone
VEGF: vascular endothelial growth factor
VEGFR2: vascular endothelial growth factor receptor
fsh: follicle stimulating hormone
